# Chemical Characterization and Antitumor Activities of Polysaccharide Extracted from *Ganoderma lucidum*

**DOI:** 10.3390/ijms15059103

**Published:** 2014-05-22

**Authors:** Zengenni Liang, Youjin Yi, Yutong Guo, Rencai Wang, Qiulong Hu, Xingyao Xiong

**Affiliations:** 1 College of Horticulture and Landscape, Hunan Agricultural University, Changsha 410128, China; E-Mails: enni_007@163.com (Z.L.); huqiulongnet@126.com (Q.H.); 2 College of Food Science and Technology, Hunan Agricultural University, Changsha 410128, China; E-Mail: 13875946008@163.com; 3 State Key Laboratory of Sub-Health Intervention Technology, State Administration of Traditional Chinese Medicine, Changsha 410128, China; E-Mail: gytyjj@126.com

**Keywords:** human colon cancer cells, apoptosis, mitochondria, caspase, MAPK

## Abstract

*Ganoderma lucidum* polysaccharide (GLP) is a biologically active substance reported to possess anti-tumor ability. Nonetheless, the mechanisms of GLP-stimulated apoptosis are still unclear. This study aims to determine the inhibitory and apoptosis-inducing effects of GLP on HCT-116 cells. We found that GLP reduced cell viability on HCT-116 cells in a time- and dose-dependent manner, which in turn, induced cell apoptosis. The observed apoptosis was characterized by morphological changes, DNA fragmentation, mitochondrial membrane potential decrease, S phase population increase, and caspase-3 and -9 activation. Furthermore, inhibition of c-Jun *N*-terminal kinase (JNK) by SP600125 led to a dramatic decrease of the GLP-induced apoptosis. Western blot analysis unveiled that GLP up-regulated the expression of Bax/Bcl-2, caspase-3 and poly (ADP-ribose) polymerase (PARP). These results demonstrate that apoptosis stimulated by GLP in human colorectal cancer cells is associated with activation of mitochondrial and mitogen-activated protein kinase (MAPK) pathways.

## 1. Introduction

Colorectal cancer (CRC) is a major public health problem that arises from the epithelium of the mucous membrane within the colon. It is the third most common cancer globally with a high mortality rate and an increasing incidence rate among young adults under 50 years of age [1,2]. Every year an estimated 8 million new cases of colorectal cancer are diagnosed, however, clinical trials with adjuvant chemotherapy have shown little improvement in survival rates, as well as the severe side effects [3]. In 2012, an estimated 143,460 colon and rectal cancer cases were diagnosed and 51,690 patients died from colorectal cancer in the United Stated [4].

Apoptosis is an ordered and orchestrated mechanism of cellular action that can be engaged by a range of cellular insults in physiology and pathology [5]. Neoplasm is a cell proliferative disorder and deregulation of apoptosis with multiple causes including both genetic and environmental factors [6]. Currently, radiotherapy, chemotherapy and immune regulation are considered as treatments that exert their effects by triggering cell apoptosis. Effective apoptotic activities induced by remedy have become a hallmark in cancer prevention [7]. Studies have shown that the enhancement of apoptosis following DNA damage prevents the development of colorectal cancer, which may prove to be a key mechanism of cancer treatment [8].

*Ganoderma lucidum* polysaccharide (GLP) from the extract of a basidiomycete fungus has been shown to significantly suppress tumorigenesis, viability, invasion and metastasis of several cancer cell lines by its cytotoxic and anti-angiogenic effects *in vitro* and *in vivo* [9]. GLP is also believed to have anti-tumor effects mediated by boosting host immune cells including macrophages, lymphocytes, natural killer cells and dendritic cells [10,11]. Furthermore, substantial reports support the belief that GLP possesses non-toxicity and reduced toxicity-related conditions due to chemotherapy/radiotherapy on normal cells [12]. These studies disclose that GLP may potentially serves as a chemopreventive agent for cancer therapy. To date the molecular mechanisms in GLP-induced HCT-116 cell apoptosis have not been characterized. This research investigated the effects of GLP on inhibitory and apoptotic reactions towards HCT-116 human colon cancer cells *in vitro*. Additionally, the regulation of GLP-induced apoptosis involving mitochondria, caspases and mitogen-activated protein kinase (MAPK) was explored.

## 2. Results and Discussion

### 2.1. Yield, Purity and Characterization of Polysaccharide

**Figure 1.**
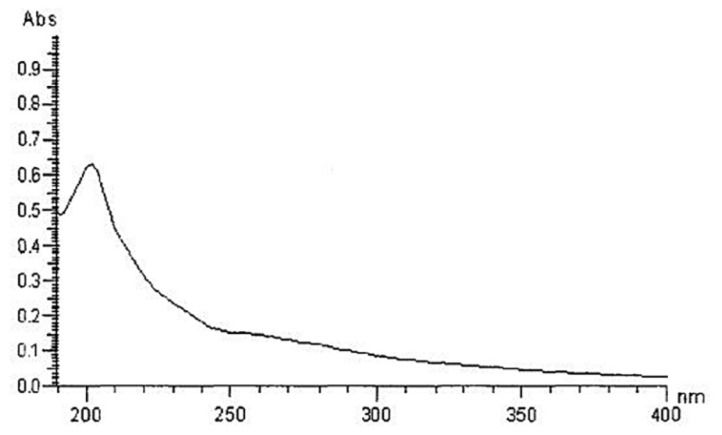
The UV spectra of polysaccharide fractions of *Ganoderma lucidum*.

The yield, based on weight of the lyophilized crude water-soluble polysaccharide, was about 3.08%. Figure 1 showed that polysaccharide fractions had no absorption at 260 and 280 nm in the UV spectrum, suggesting the absence of nucleic acid and protein. The total carbohydrate content and uronic acid analyses found that crude extract GLP was an acidic polysaccharide containing 89% of total carbohydrate and 11% of uronic acid. The molecular weight composition of the polysaccharide is as follows: 10–30 kDa, 32.1%; 30–50 kDa, 21.8%; and >50 kDa, 46.1%. The polysaccharide consists of arabinose, galactose, cellose and glucose in the molar ratios of 11:3:1:3.

**Figure 2.**
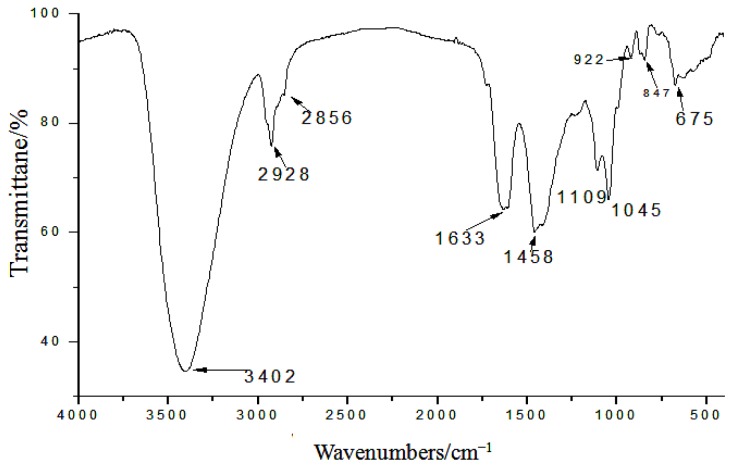
Infrared spectroscopy of *Ganoderma lucidum* polysaccharide (GLP).

IR spectra of GLP are depicted in Figure 2. The obvious polysaccharide at 3402 cm^−1^ was due to O–H stretching vibration. The spectrum also showed an intense vibration and a water-related absorbance at 1633 cm^−1^. The peak-height at 1458 cm^−1^ was attributed to C–H bending vibration. The absorption band towards 1109 cm^−1^ suggested that the peak was related to the stretching vibration of C–O. The absorption peak at 870 cm^−1^ was assigned to α-type glycosidic linkages in the polysaccharide. Typical characteristic peaks were observed at both 847 and 922 cm^−1^ for α-1,4-D-glucan [13]. Together with the high positive value of the specific rotation, the IR spectrum indicated the presence of α-glycosidic linkages in the GLP.

### 2.2. Effects of Ganoderma lucidum Polysaccharide (GLP) on HCT-116 Cell Viability

**Figure 3.**
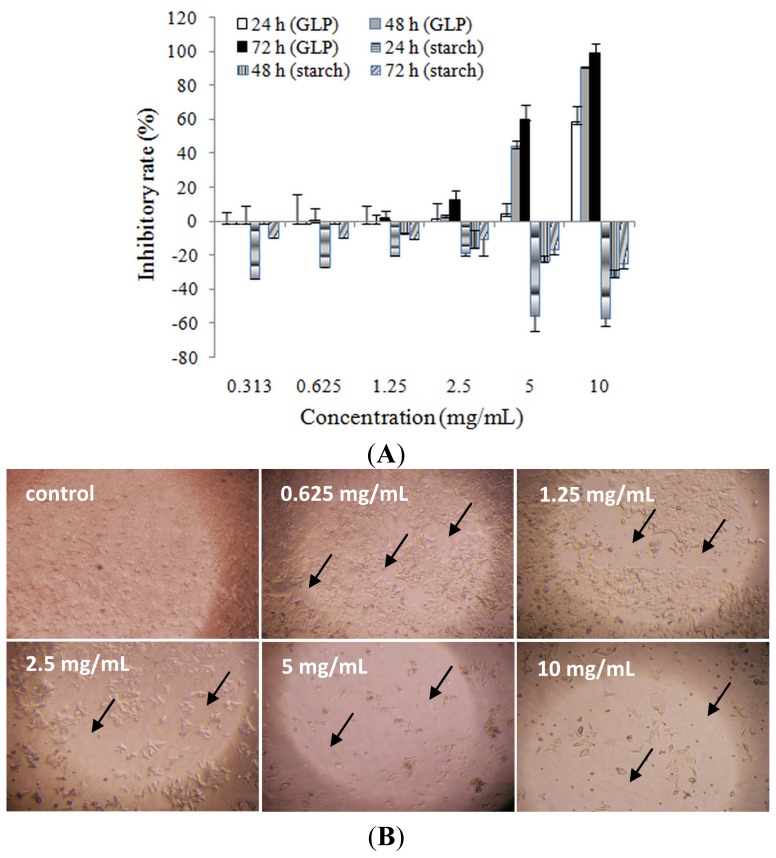
Cytotoxicity of GLP on HCT-116 cells: (**A**) GLP suppressed the cell viability of HCT-116. Inhibitory rate was measured by MTT method. Starch-incubated cells were applied as control. Data represent means ± SD of three independent experiments; and (**B**) Morphological changes in HCT-116 cells. After treatment with GLP, exfoliation of HCT-116 cells and naked areas were observed and captured under an inverted microscope (×100). The arrows (↑) show naked areas without cells.

Many previous reports have indicated that Chinese herbs inhibit the growth of cancer through direct reactions to cell killing and/or indirect reactions that boost immunity function on cancer cells [14]. To investigate whether GLP had cytotoxic effects, cell viability was performed by MTT assay after various concentrations of GLP (0.313, 0.625, 1.25, 2.5, 5 and 10 mg/mL) inoculation. As shown in Figure 3A, the result indicated that GLP had significant inhibitory effects on cell viability of HCT-116 cells in a dose- and time-dependent manner. The inhibitory concentration of 50% (IC_50_) for 24, 48 and 72 h were 9.25, 5.72 and 3.69 mg/mL, respectively, and this range of concentration was chosen for further study. The inhibitory activity of GLP lasted for at least 72 h. To exclude the possibility that the cytotoxicity of GLP on HCT-116 cells was applied by osmotic pressure, starch, a common polysaccharide produced by plants, was used as control. The results showed that starch (0.313–10 mg/mL) dose-dependently decreased cell growth, and 5-fluorouracil (5-FU) (50 μg/mL) dramatically suppressed cell viability (data not shown), suggesting the osmotic pressure within the range of concentrations of GLP did not obviously affect cell viability, and that GLP possessed cytotoxicity effects on HCT-116 cells. Figure 2B demonstrates that HCT-116 cells treated with GLP exhibited cellular morphological changes such as round sharp and volume reduction, and a concentration-dependent decrease in cellular number.

It is reported that Chinese herbs combined with adjuvant chemo- or/and radio-therapy may synergistically inhibit numerous cancers, significantly alleviate therapy side effects, improve quality of life, and prolong survival in cancer patients [15,16]. Our preliminary study was the first to report that the simultaneous treatment with GLP and 5-fluorouracil synergistically affects inhibitory and apoptotic effects on human colon cancer cells *in vitro* [17]. In theory, the anti-tumor effect of GLP was associated with its sub-fraction. Li *et al.* [18] reported that high molecular weight polysaccharides play an important role in cancer resistance, so GLP (molecular weight >10 kDa) were studied for their anti-tumorogenic effects on HCT-116 cells.

### 2.3. Effects of GLP on HCT-116 Cell Apoptosis

**Figure 4.**
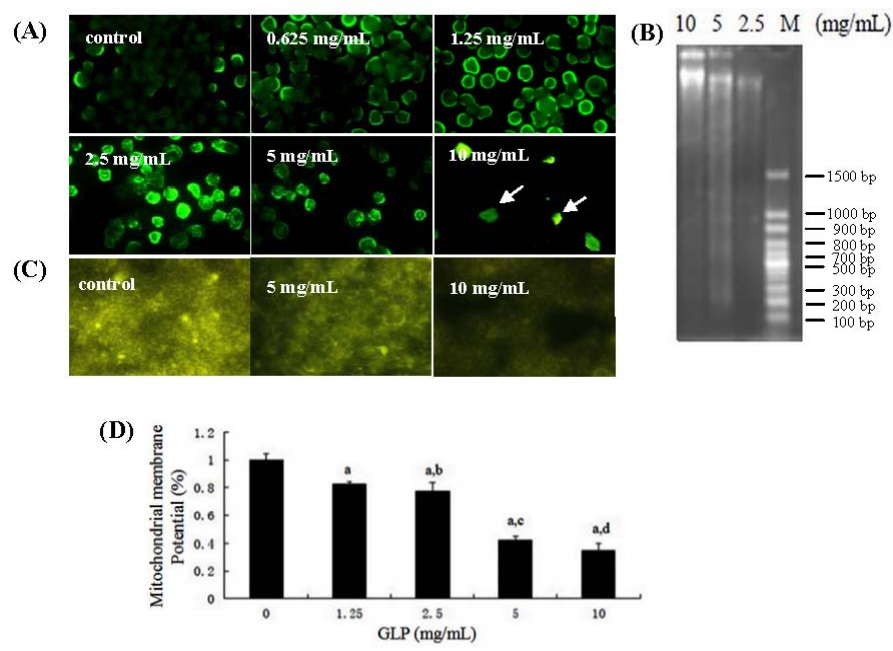
Effects of GLP on apoptosis in HCT-116 cells. (**A**) Apoptotic cells measured by Hoechst 33,258 staining after treatment with GLP for 48 h under a fluorescence microscope (×200). The arrows show cell fragments; (**B**) HCT-116 cells were exposed to the indicated concentrations of GLP for 24 h. DNA was isolated and examined on 1.2% agarose gel; (**C**) Mitochondrial membrane potential (ΔΨ_m_) was monitored by microscopy and photographed at each concentration of GLP; and (**D**) quantitative evaluation. Data are presented as mean ± SD. a, *p* < 0.01 compared with control. b, c and d, *p* < 0.01 compared with 1.25, 2.5 and 5 mg/mL GLP treatment, respectively.

As an important manner of cell death, apoptosis involves morphological changes with a series of stereotypy, DNA laddering fragments, phosphatidylserine eversion, and regulation of related factors and intracellular calcium concentration of cells [19]. DNA fragmentation has been found during GLP-mediated apoptosis in human leukemia HL-60 cells [20]. In the current study, we revealed that GLP directly induced HCT-116 apoptosis *in vitro*, as demonstrated by morphology, DNA gel electrophoresis and mitochondrial membrane potential. As shown in Figure 4A, cytoplasmic diffusion and cellular membrane integrity were observed in untreated cells. Clear signs of decreased cellular integrity accompanied by increased Hoechst 33,258 staining, are effects seen at GLP concentrations as low as 0.625 mg/mL after 48 h. Cellular nuclei were clearly fragmented by increasing GLP concentration up to 10 mg/mL. DNA ladder fragmentation was observed using agarose gel electrophoresis in human colon cancer cells incubated with 2.5 to 10 mg/mL GLP (Figure 4B). In Figure 4C,D, control cells appeared bright yellow with R123 staining. The R123 fluorescence of HCT-116 cells treated with GLP was weaker, and these effects were dose-dependent, indicating that those cells had damaged mitochondrial membrane integrity. Since these results in addition to 50 μg/mL 5-FU can result in apoptosis on HCT-116 cells, it is implicated that GLP inhibits cell viability via an apoptotic pathway in human colon cancer cells.

### 2.4. Detection of Cell Cycle and Apoptosis by Flow Cytometry

**Figure 5.**
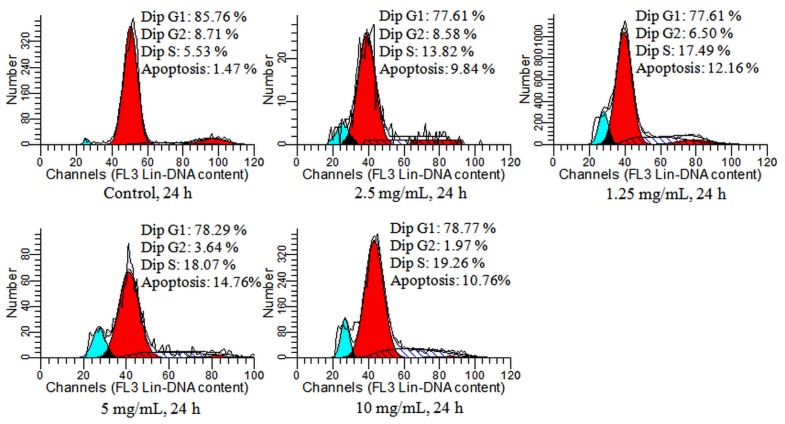
Flow cytometry analysis of GLP-treated HCT-116 cells. Cells were incubated with GLP at various concentrations (1.25–10 mg/mL) for 24 h and then were harvested for quantifying apoptosis and cell cycle phase by flow cytometry (Propidium Iodide (PI) staining). Apoptosis and S phase cell populations of GLP-treated group are significantly higher than in the control. The blue peak = apoptosis; the first red peak = G1; the second red peak = G2; hatched peak = S.

GLP-mediated apoptosis was confirmed by flow cytometry in HCT-116 cells. As shown in Figure 5, after 24 h of GLP exposure, apoptosis rates were boosted from 9.84% to 14.76% at 1.25 and 10 mg/mL, respectively, with a maximum 10-fold apoptosis rate compared to the untreated contrast at 1.47% (*p* < 0.01).

Cell cycle including at least 3 checkpoints (G_1_/S, intra-S-phase and G_2_/M) has an essential role in regulating proliferation [21]. These checkpoints can enhance cell survival by limiting mutagenic events and decreasing abnormal heritable genetic changes following DNA damage [22]. Deregulated cell viability and suppressed cell death together provide the underlying opportunity for neoplastic progression [23]. Some anti-cancer agents can act as cell cycle blockers to interfere and switch cell cycle progression at a specific stage in cancer cells [24].

Figure 5 also shows that HCT-116 cells incubated with 1.25, 2.5, 5 and 10 mg/mL GLP were significantly increased in S phase (13.82%, 17.49%, 18.07% and 19.26%, respectively). Compared with the control group (5.53%), the percentage of GLP-treated cells in S phase significantly increased which was accompanied by a decrease in G_1_ and G_2_/M phase. The result was consistent with our previous results in a synergy trial of GLP and 5-FU [17]. Interestingly, GLP could arrest different cell cycle phases in other cancer cells. GLP has been reported to increase the percentage of G_0_/G_1_ in S180 ascitic tumor-bearing mice [25] and human leukemia THP-1 cells [26], indicating that the mechanisms of GLP-mediated apoptosis associated with cell cycle arrest are different.

### 2.5. Effects of GLP on the Mitogen-Activated Protein Kinase (MAPK) Pathway

**Figure 6.**
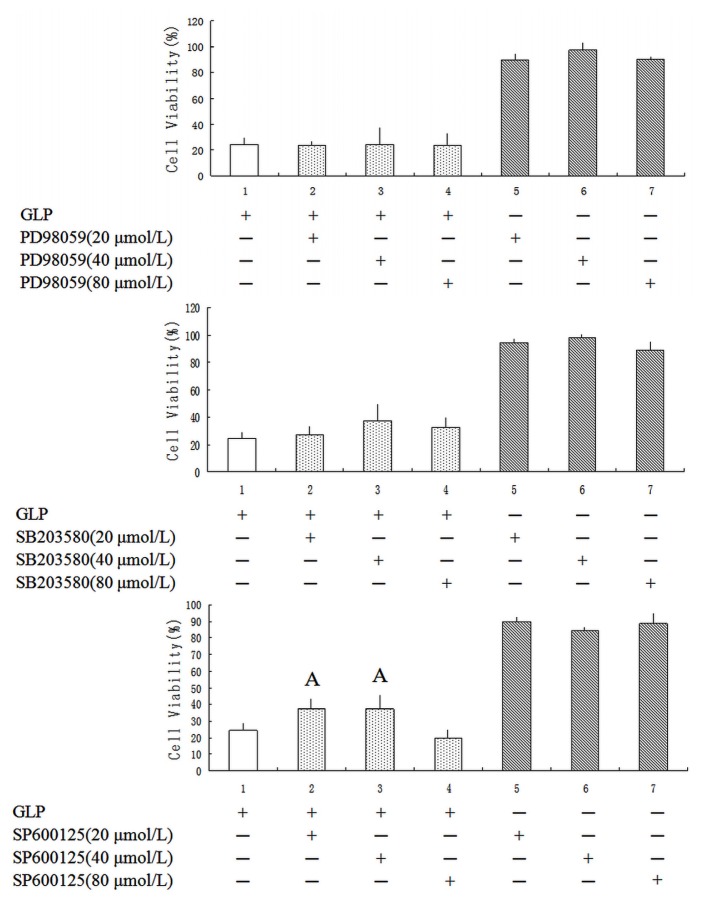
Effects of mitogen-activated protein kinase (MAPK) inhibitors on GLP-induced HCT-116 cell death. Cells were treated in the absence or presence of different MAPK-specific inhibitors, 1 h prior to the addition of GLP, and then incubated in 5 mg/mL GLP for 12 h. Cells were harvested to determine the percentage of viable cells as described in the Experimental section. A, *p* < 0.05 compared with only 5 mg/mL GLP treatment group. Data expressed as mean ± SD.

Components of the c-Jun *N*-terminal kinase (JNK), extracellular signal-regulated kinase (ERK) and p38 MAPK pathways are known to regulate the cell cycle, differentiation, growth and cell apoptosis [27]. The aim here was to determine whether GLP induced activation of ERK1/2, JNK and p38 in HCT-116 cells. As presented in Figure 6, the inhibitors of PD98059 and SB203580 did not affect cell growth. Twenty and 40 μmol/L SP600125 were able to partially protect human colon cells from GLP-treated cells with a significant decrease in cell viability (*p* < 0.05). This finding suggests that GLP could up-regulate JNK expression via the MAPK pathway to induce apoptosis in HCT-116 cells, whereas p38 and the ERK and MAPK pathway do not contribute to this process in HCT-116 cells.

### 2.6. Effects of GLP on Apoptosis-Related Proteins

Mitochondria play a key role of mediator, and are an appropriate target for therapeutic agents for active control of cell death programs [28]. Once mitochondrial dysfunction, embodying such events as the opening of the mitochondrial permeability transition pore complex and depolarization of mitochondrial membrane potential (ΔΨ_m_) has occurred, the intrinsic mitochondrial apoptotic pathway is initiated. Subsequently, caspases are activated and cell apoptosis is irreversible [29]. Some proteins in the mitochondrial pathway are related to control of cell apoptosis. The ratio of pro-apoptotic and anti-apoptotic proteins (Bax/Bcl-2) determines susceptibility of mitochondria-mediated apoptosis; cleavage of Poly (ADP-ribose) polymerase (PARP) facilitates cellular disintegration and is an important marker of cells undergoing apoptosis [30].

**Figure 7.**
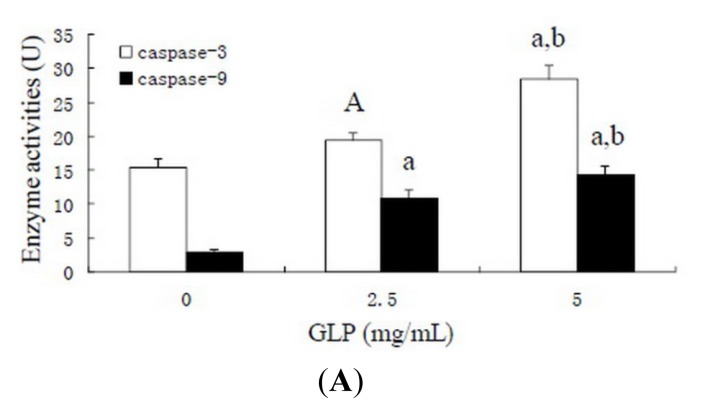

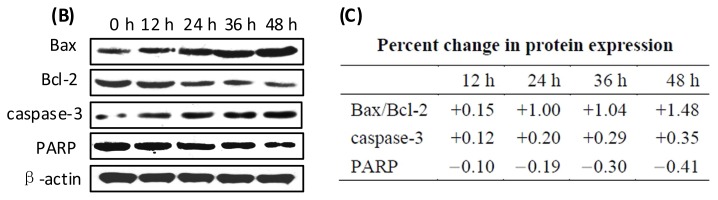
GLP induces cell apoptosis via caspase-dependent mitochondrial pathways. (**A**) Activity of caspases in GLP treated HCT-116 cells. A, *p* < 0.05, and a, *p* < 0.01 compared with control. b, *p* < 0.01 compared with 2.5 mg/mL GLP treatment; (**B**) Western blot analyses of mitochondria pathway-related proteins. All bands were compared with the β-actin band and indicated as down-regulation (−) or up-regulation (+); and (**C**) Statistical analysis for western blot analysis.

We found that loss of mitochondrial membrane potential was involved in GLP-mediated apoptosis of HCT-116 cells (Figure 4C,D). To determine the effects of the mitochondrial pathway on GLP-mediated apoptosis, caspase-3 and -9 activities were assessed. After HCT-116 cells were incubated with GLP for 24 h, caspase-3 and -9 activities increased in a concentration-dependent manner (Figure 7A). These results suggest that caspases play a critical role in GLP-induced HCT-116 cell apoptosis. To further define HCT-116 apoptosis associated with caspase-dependent signal pathways, Western blotting was used to examine expression of Bax, Bcl-2, caspase-3, and PARP protein. As shown in Figure 7B, exposure of HCT-116 cells to 5 mg/mL GLP led to increasing levels of apoptotic Bax and caspase-3 in a dose-dependent manner. The expression of the anti-apoptotic Bcl-2 and PARP proteins were dose-dependently reduced by GLP exposure. The Bax:Bcl-2 ratio was increased from 0.15 to 1.48. These results suggest that the caspase-dependent mitochondrial pathway performs a crucial function in GLP-induced apoptosis in HCT-116 cells. This is in agreement with other studies [31].

## 3. Experimental Section

### 3.1. Materials

*Ganoderma lucidum* was provided by the State Key Laboratory of Sub-health Intervention Technology, State Administration of Traditional Chinese Medicine (Changsha, China). Colon cancer cell line HCT-116 was obtained from Institute of Basic Medical Sciences (Beijing, China). Cells were grown in DMEM high glucose (Gibco, Grand Island, NY, USA) supplemented with 10% FBS (Gibco) under 5% CO_2_ at 37 °C. MTT, Hoechst 33258, caspase-3 and -9 activity assay kits were purchased from Beyotime, China. 5-Fluorouracil (5-FU, >99% purity) was purchased from Shanghai Bangcheng Chemical Co., Ltd. (Shanghai, China). Inhibitors of JNK MAPK (SB203580), p38 MAPK (SP600125) and ERK MAPK (PD98059) were purchased from Biovision (Milpitas, CA, USA). The caspase-3, PARP, Bax, Bcl-2 and β-actin primary antibodies were purchased from Proteintech Group, USA; all other chemicals used were analytical grade unless stated otherwise.

### 3.2. Isolation and Purification of GLP

Slices of *Ganoderma lucidum* were dried at 60 °C and ground to a fine powder. The powder samples were refluxed in three volumes of methanol/chloroform solvent (1:2, *v*/*v*) and 80% ethanol three times for six-hour duration each time, respectively. After solution removal, dried residues were suspended in distilled water (residues:water = 1:15, *v*/*v*) following by vacuum filtration three times at 95 °C and 6 h for each time. The combined filtrates were concentrated with a vacuum rotary evaporator at 50 °C. The concentrated solution was ethanol precipitated and then deproteinized using a Sevage assay. The extracts were purified using DEAE-Cellulose (DE 52), dialyzed, concentrated and freeze-dried. Ten milligram per milliliter GLP was ultrafiltered through membranes with molecular weight cut-off (MWCO) of 50, 30, 10 and 3 kDa (Millipore, Billerica, MA, USA) in an Amicon 8200 stirred cell (Millipore). The filtrate was concentrated and freeze-dried again. The dried powder was dissolved in DMEM culture medium (high glucose) supplemented with 10% FBS to get a stock solution of 10 mg/mL, passed through 0.22 μm filter and stored at 4 °C for subsequent analysis.

### 3.3. Chemical Properties

The yield of crude polysaccharide was measured as a percentage of the total weight of sample used. The polysaccharide content was calculated by the phenol-sulfuric acid method. After water-soluble polysaccharides were purified, GLP was ultrafiltered using membranes (Biomax-500, Biomax-300, Ultracel PL-100 and Ultracel PL-30) with MWCO of 3–50 kDa. The distribution of molecular weight was analyzed by weighing five freeze-dried fractions. Protein and nucleic acids were analyzed by UV spectrum. After GLP was hydrolyzed with 1 mol/L H_2_SO_4_ for 8 h at 100 °C, the monosaccharide components were analyzed by high-performance anion exchange chromatography (HPAEC). The compounds of polysaccharide were identified by ultraviolet and infrared spectra.

### 3.4. Cell Viability Assay

MTT assay was used to evaluate the antiproliferative effects of GLP in HCT-116 cells. Cells were seeded at 3 × 10^4^ cells/well in 96-well tissue culture plates and allowed to grow for 12 h. The medium was replaced with fresh medium containing different concentrations of GLP (0.625 to 10 mg/mL) for 24, 48 and 72 h. At each time point, cells were added with 20 μL MTT (5 mg/mL) solution and continued to incubate for 4 h at 37 °C. The resolution was removed, and 150 μL DMSO was added to dissolve the formazan. Cells with starch (0–10 mg/mL) treatments were used as control and 50 μg/mL 5-FU was applied as positive control. Plates were observed to compare the colors of the resolution in the wells. Absorbance was read at 492 nm with Multiskan Microplate Reader (MK3, Thermo, Waltham, MA, USA).

### 3.5. Hoechst 33258 Staining

Apoptosis was determined by staining cells with Hoechst 33258 staining. Cells in 25 cm^2^ culture flasks were incubated with the indicated amounts of GLP or 50 μg/mL 5-FU treated cells. Cells were washed with cold PBS and fixed with 2.5% glutaraldehyde for 10 min. After being washed with PBS twice, cells were incubated with 5 μg/mL Hoechst 33258 (Beyotime, Haimen, China) for 15 min at room temperature and then visualized under a fluorescence microscopy (Olympus, Tokyo, Japan).

### 3.6. Measurement of DNA Fragmentation

Apoptosis of cancer cells was detected for their DNA degradation by DNA gel electrophoresis analysis. Cells were treated with GLP (2.5, 5 and 10 mg/mL) or 5-FU (50 μg/mL) for 24 h. After two washes with PBS, cells were resuspended in 5 mL of 70% ethanol for 4 h at −20 °C and centrifuged at 1000× *g* for 5 min at 4 °C. The precipitate was dissolved in 40 μL phosphate-citric acid buffer, pH 7.8, and incubated for 45 min at room temperature with intermittent shaking. The solution was centrifuged at 1500× *g* for 5 min. The supernatant was collected using a new tube, mixed with 3 μL of 1 mg/mL RNaseA and 3 μL of 0.25% NP40, and then incubated at 37 °C for 30 min. DNA fragmentation was then analyzed by gel electrophoresis on a 1.2% agarose gel.

### 3.7. Mitochondrial Membrane Potential Analysis

After treatment with GLP (0, 1.25, 2.5, 5 and 10 mg/mL) for 24 h, cells were washed with PBS twice and incubated with 8 μM rhodamine123 (R123) at 37 °C for 30 min in the dark. Cells were washed two times again. Cells treated with 50 μg/mL 5-FU served as a positive control. The intensity of fluorescence was observed under a fluorescence microscope (Olympus, Tokyo, Japan) and measured using a fluorometric plate reader (Corning, Corning, NY, USA) equipped with a Scientific Varioskan Flash (Thermo) at an excitation wavelength of 495 nm and an emission wavelength of 530 nm.

### 3.8. Caspase Activity Assay

Caspase-3 and -9 activities in cells were determined by the cleavage of colorimetric caspase substrates, Ac-DEVD-*p*NA and Ac-LEHD-*p*NA, using the caspase-3 and -9 activity kits (Beyotime, China). In brief, HCT-116 cells were treated with 0, 2.5 and 5 mg/mL GLP for 24 h in 6-well plates. After centrifugation, cells were lysed on ice for 15 min. The cells were centrifuged at 10,000× *g* for 20 min at 4 °C and the supernatants were then transferred to 96-well microtiter plates. Assays were determined by incubating 10 μL protein of cell lysate in 80 μL reaction buffer (1% NP-40, 20 mM Tris–HCl, pH 7.5, 137 mM NaCl and 10% glycerol) and 10 μL of 2 mM substrate. After incubation for 2 h at 37 °C, the absorbance of cleavage (*p*NA) was quantified with Multiskan Microplate Reader (MK3, Thermo, USA) at 405 nm.

### 3.9. Flow Cytometry

Cells (1 × 10^6^) were seeded into 6-well culture plates. After treatment with GLP for 24 and 48 h, cells were washed three times with PBS. The cells were then fixed overnight in 70% ethanol. After overnight incubation at 4 °C, cells were rehydated in PBS for 30 min and subsequently stained with dye liquid (1 g/L sodium citrate, 1 mg/L RNase A, 50 mg/L propidium iodide, 10 g/L Triton X-100) at 4 °C for 30 min. 50 μg/mL 5-FU was used as a positive control. The apoptosis rate and cell cycle was analyzed by an FC500 flow cytometer (Beckman Coulter Inc., Miami, FL, USA).

### 3.10. MAPK Inhibitors Assay

To investigate the effects of MAPK inhibitors on eliciting apoptotic GLP-treated cancer cells, MAPK inhibitors including PD98059 (ERK specific inhibitor), SB203580 (JNK specific inhibitor) and SP600125 (p38 specific inhibitor) at concentrations of 20, 40 and 80 μmol/L were introduced to cells and cultivated for 1 h after seeding cells (3 × 10^3^ cells/well) in 96-well culture plates for 24 h. Subsequently, 5 mg/mL GLP was added, and samples were incubated for 24 h at 37 °C. At the end of the assay, the cells were estimated using the Multiskan Microplate Reader at 490 nm.

### 3.11. Western Blot Analysis

Cells were treated with 5 mg/mL GLP for 0, 12, 24, 36 and 48 h, and then lysed in RIPA lysis buffer (Applygen, Beijing, China) (50 mM Tris, pH 7.4, 150 mM NaCl, 1% Triton X-100, 1% sodium deoxycholate, 0.1% SDS, sodium orthovanadate, sodium fluoride, EDTA, leupeptin) on ice for 30 min. After centrifugation at 12,000 rpm, proteins were measured by the Bio-Rad Bradford assay with bicinchoninic acid (BCA) as the standard, separated by a 10%–12% SDS-PAGE and transferred onto 0.45 μm polyvinylidene fluoride (PVDF) membranes (Millipore, Billerica, MA, USA) according to the methods as described [32]. The membranes were blocked in 5% non fat milk in TBS (10 mmol/L Tris–HCl, pH 7.5 and 150 mmol/L NaCl) containing 0.1% Tween (TBST) for 1 h at room temperature followed by incubation overnight at 4 °C with the chosen primary antibodies, including β-actin (1:4000), caspase-3 (1:500), PARP (1:800), Bax (1:800) and Bcl-2 (1:800). The membrane was washed with TBST three times, and the proteins were labeled using horseradish peroxidase-conjugated goat anti-rabbit or anti-mouse secondary antibody (1:3000) and enhanced using ECL system (Thermo Scientific Pierce, Waltham, MA, USA). The bands were visualized by exposure on X-OMAT BT films (Fujifilm, Tokyo, Japan). The films were scanned and densitometric analyses were performed using ImageJ software (National Institutes of Health, Bethesda, MD, USA).

### 3.12. Statistical Analysis

All calculations were performed with SPSS 18.0 for Windows (SPSS, Chicago, IL, USA). All data are expressed as means ± SD. The significance of differences among groups was analyzed using one-way ANOVA followed by Bonferroni’s test for multiple-comparison or Student’s *t* test, with significance at *p* < 0.05 and extreme significance at *p* < 0.01.

## 4. Conclusions

In summary, our study provides significant insight into the cytotoxicity and apoptosis inducing ability of GLP in human colon cancer HCT-116 cells. Raising the Bax/Bcl-2-ratio triggered the apoptotic process though mitochondria-mediated caspase-dependent intrinsic pathway, involving depolarization of mitochondrial membrane. With the addition of GLP treatment, the MAPK signaling pathway and cell cycle arrest at S phase is also attributed to apoptosis in HCT-116 cells. These data provide evidence for the possible mechanism of GLP-mediated apoptosis. However, other signaling pathways such as TNF, Fas, P53 pathway might also be involved in GLP-mediated apoptosis and it is not yet clear whether there is a similar tendency in animal models or clinical studies.
